# Neck Dissection with Harmonic Instruments and Electrocautery: A Systematic Review

**DOI:** 10.1055/s-0044-1779010

**Published:** 2024-02-05

**Authors:** Rogério Aparecido Dedivitis, Leandro Luongo de Matos, Mario Augusto Ferrari Castro, Sílvia Migueis Picado Petrarolha, Luiz Paulo Kowalski

**Affiliations:** 1 Department of Head and Neck Surgery, Faculty of Medicine, University of São Paulo, São Paulo, SP, Brazil; 2 Department of Head and Neck Surgery, Hospital das Clínicas, Faculty of Medicine, University of São Paulo, São Paulo, SP, Brazil; 3 Instituto do Câncer do Estado de São Paulo (ICESP), SP, Brazil; 4 Universidade Metropolitana de Santos, Medicine School, Santos, SP, Brazil

**Keywords:** neck dissection, surgical hemostasis, squamous cell carcinoma, meta-analysis

## Abstract

**Introduction** The harmonic scalpel (HS) is a technique introduced to reduce blood loss and intraoperative time during neck dissection (ND).

**Objective** To compare the results of HS with traditional hemostasis in ND through a systematic review and metanalysis.

**Methods** A computer-based strategy of systematic literature survey included research in the MEDLINE, EMBASE, and Cochrane Library databases from January 2007 up to August 2022. The survey strategy employed was [harmonic scalpel OR ultrasonic scalpel] AND neck dissection.

**Results** There were 61 articles identified that addressed the use of HS in patients undergoing ND. From those, 10 randomized clinical trials were selected, comprising 264 cases of ND using HS and 262 cases of ND without HS.

**Conclusion** The use of HS for ND significantly reduces the operative time, intraoperative bleeding, volume of draining fluid, and the number of ligatures.

## Introduction

Neck dissection (ND) is a part of surgical therapy for head and neck cancer. During ND, control of hemostasis is essential. Additionally, several studies have shown that operative time and amount of blood loss are related to clinical outcomes and complication rates. 1 2 3 4 Many techniques have been introduced to reduce blood loss and intraoperative time during ND, including monopolar and bipolar cauterization, radiofrequency ablation and hemoclips. 5

Since its introduction in 1990, the harmonic scalpel (HS, Harmonic, Ethicon Endo-Surgery, Cincinnati, Ohio, EUA) has become popular in head and neck surgery. 6 Ultrasonic energy is used to cut and coagulate soft tissues. Its mechanism of action is based on the conversion of electrical energy into mechanical energy (ultrasonic vibration). This technique has shortened the operative time in thyroidectomy compared to the conventional one. 7 It has also been regularly used to perform other procedures such as tonsillectomy, glossectomy and parotidectomy.

Koh et al. studied the use of HS in ND and compared it with the electrocautery technique, reporting shorter operative time and reduced blood loss with HS. 8 Since then, a small number of references in the literature have defended the use of HS for performing ND. Additionally, there are very limited data comparing HS and conventional electrosurgical techniques. 9

The objective of this systematic review is to compare the results of HS with traditional hemostasis in ND through a metanalysis.

## Methods

Identification and selection of the studies happened through a computer-based strategy of literature survey employed to perform the systematic review of the available evidence. It included the research in MEDLINE, EMBASE, and the Cochrane Library databases from January 2007 up to August 2022. Titles and abstracts reporting the outcome of HS and conventional technique in ND were selected. The survey strategy employed was: [harmonic scalpel OR ultrasonic scalpel] AND neck dissection. References from the selected articles were evaluated as well. Two authors evaluated independently the articles. In case of disagreement, a third author performed the eventual decision.

The inclusion and exclusion criteria were patients diagnosed with head and neck who underwent ND. The article must have compared the outcomes of the HS and the conventional hemostasis technique in ND. Only randomized prospective studies were considered regardless its language. The paper must contain data enough for evaluating the outcomes of interest.

The risk factors evaluated were operative time (in minutes), intraoperative blood loss (mL), total suction drain output (mL), time of drain use (days), number of ligatures used, number of lymph nodes dissected, pain (virtual analogue scale, VAS in postoperative 24 hours and 48 hours), and hospital stay (days).

The measures of each risk factor were expressed through absolute values and analyzed by means of the difference of the absolute risk, under the 95% confidence interval (CI). Inconsistence among clinical trials were evaluated through the chi-square heterogeneity test (Chi ^2^ ) and quantified using the I2 test. The chi-square test shows the percentage of total variation across studies caused by heterogeneity and was used to judge the degree of consistency evidence obtained. Values lower than 20% were considered presenting low heterogeneity; from 20 to 50%, with moderate heterogeneity and higher than 50%, with high heterogeneity.

## Results

**Fig. 1:**
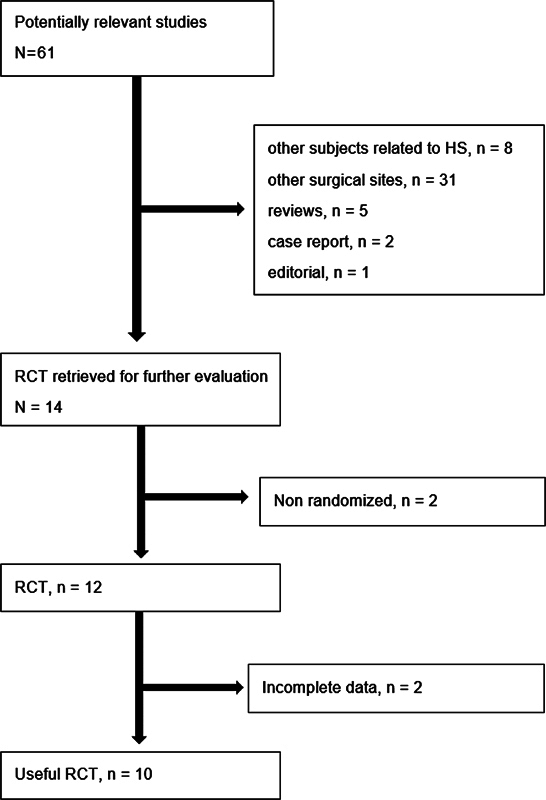
Flow chart of the bibliographic research.

Our literature review identified 61 articles that addressed the use of HS and conventional technique in patients undergoing ND, 47 of which were excluded because they did not meet the inclusion criteria. The remaining 14 articles, consisting of clinical trials, were evaluated; 2 were excluded for not being randomized, and 4 had incomplete data for the metanalysis (absence of information on standard deviations). However, after contacting the respective authors, they provided the necessary data referring to 2 studies, allowing them to be included. Thus, 10 articles were selected. Figure 1 shows the flowchart of retrieved and excluded studies and lists the reasons for their exclusion. The final selection was of 10 studies comprising a total of 264 cases of ND with HS and 262 cases without.

**Fig. 2:**
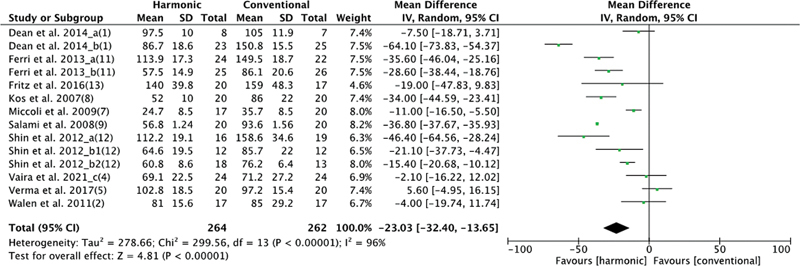
Forest plot for surgical time. Operative time with HS was significantly shorter than with conventional hemostasis.

There were 10 studies that evaluated operative time, with 264 patients in the HS group and 262 in the conventional hemostasis group. When these studies were quantitatively combined, the time was significantly shorter in the HS group: mean difference: −23.03 (−32.4, −13.65); I2 = 96%; *p* = 0.00001 ( Figure 2 ).

**Fig. 3:**
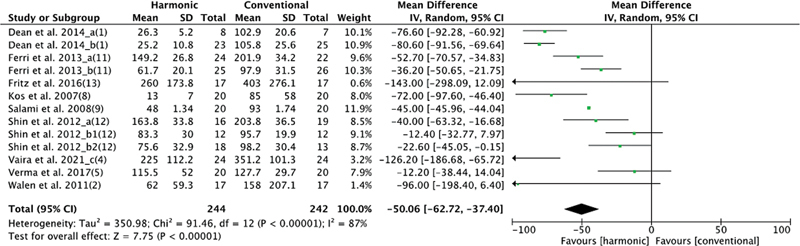
Forest plot for intraoperative blood loss: the bleeding volume was significantly lower in the HS group.

As for intraoperative bleeding, 9 studies studied this variable, with 244 patients in the HS group and 242 in the conventional hemostasis group. When these studies were quantitatively combined, the blood loss was lowest in the group using HS: mean difference: −50.06 (−62.72, −37.4); I2 = 87%; *p* = 0.00001 ( Figure 3 ).

**Fig. 4:**
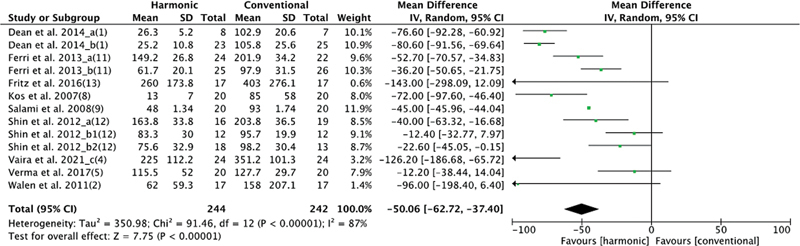
Forest plot for total suction drain output. The drain volume was significantly smaller in the HS group.

There were 10 studies evaluating the amount of drainage, with 261 patients in the HS group and 262 in the conventional hemostasis group. When these studies were quantitatively combined, there was a lower total volume of drainage fluid for the group using HS: mean difference: −247.78 (−303.76, −191.8); I2 = 100%; *p* = 0.00001 ( Figure 4 ).

**Fig. 5:**
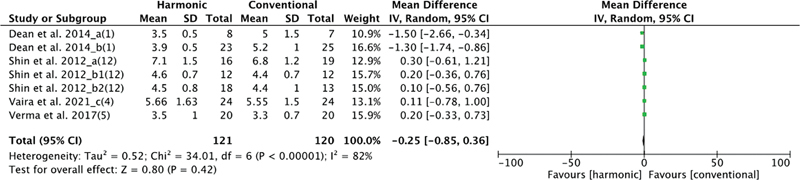
Forest plot for time of drain use (no significant difference).

The duration of drain use was evaluated in 4 studies, with 121 patients in the HS group and 120 in the conventional hemostasis group. When these studies were quantitatively combined, there was no significant difference when comparing hemostasis methods: mean difference: −0.25 (−0.85, 0.36); I2 = 82%; *p* = 0.0001 ( Figure 5 ).

**Fig. 6:**
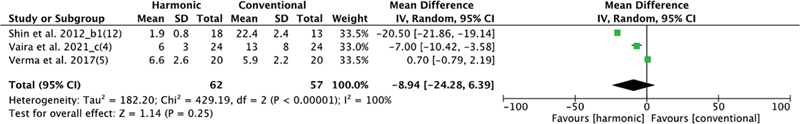
Forest plot for number of ligatures (no significant difference).

There were 3 studies that evaluated the number of suture ligations performed during ND, with 62 patients in the HS group and 57 in the conventional hemostasis group. When these studies were quantitatively combined, there were fewer ligatures in the group using the HS: mean difference −8.94 (−24.28, 6.39); I2 = 100%; *p* = 0.0001 ( Figure 6 ).

**Fig. 7:**
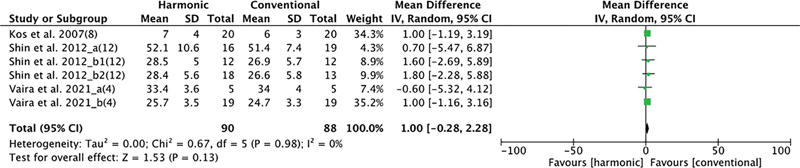
Forest plot for number of lymph nodes dissected (no significant difference).

The number of dissected lymph nodes was evaluated by 3 studies, with 90 patients in the HS group and 88 in the conventional hemostasis group. When these studies were quantitatively combined, there was no significant difference between hemostasis methods: mean difference −0.29 (−0.29, 2.28); CI = 0%; *p* = 0.98 ( Figure 7 ).

**Fig. 8:**
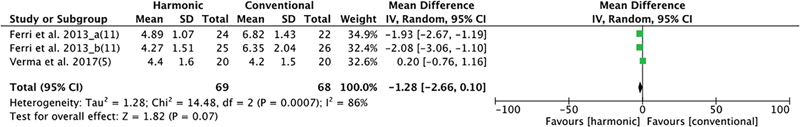
Forest plot for pain at 24 hours (no significant difference).

**Fig. 9:**
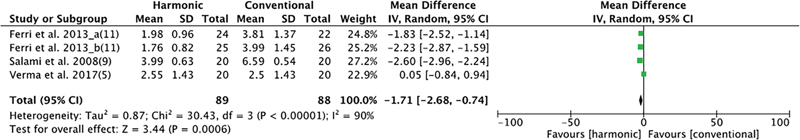
Forest plot for pain at 48 hours (no significant difference).

There were 2 and 3 retrospectively evaluating the 24-hour and 48-hour postoperative local pain scale with 69 patients in the HS group and 68 in the 24-hour conventional hemostasis group and 89 patients in the HS group and 88 in the conventional hemostasis group for 48 hours. When these studies were quantitatively combined at each period, there was no significant difference between hemostasis methods ( Figures 8 and 9 ).

**Fig. 10:**
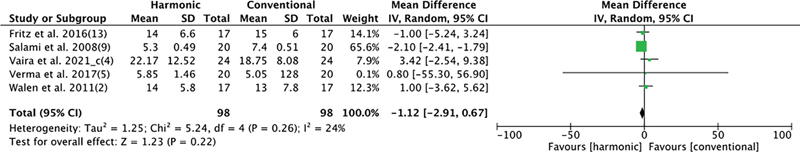
Forest plot for hospital stay (no significant difference).

Finally, 5 studies evaluated hospital stay. When these studies were quantitatively combined, there was no significant difference between the methods studied: mean difference −1.12 (−2.91, 0.67); I2 = 24%; *p* = 0.22 ( Figure 10 ).

## Discussion

The ND is well-established procedure for locoregional control in head and neck cancer treatment. The introduction of new technology-based operative techniques can mitigate the negative consequences of this operation.

Different cutting and coagulation methods aim to reduce complications, shorten operative time, improve surgeon's comfort, and reduce morbidity. Control of hemostasis is essential in neck dissection. The vibration of the active HS lamina breaks hydrogen bonds and defragments proteins, which seals smaller vessels. Furthermore, friction creates secondary heat that denatures the protein. Separation of the anatomical planes occurs at temperatures between 60 and 80°C. 4 Few studies have compared the effectiveness of HS with traditional techniques for performing ND; however, its use has become progressively more popular because the demonstration of its effectiveness and safety. 5

There is controversy among the different studies regarding the reduction of surgical (and anesthetic) time with the use of HS. Vaira et al. 4 found shorter surgical time in the conventional technique, but there was an increased time in the first cases with HS due to the learning curve. Walen et al., 3 Verma et al., 5 and Fritz et al. 10 found similar results. In contrast, Dean et al., 1 Ferri et al., 11 and Shin et al. 12 reported a shorter operative time using HS. Thus, the metanalysis showed this technique's benefit. The reduced duration is due to the lower need for clotting of small blood vessels and an optimized vision in the bloodless operative field.

Blood loss during ND can be reduced because of shorter operating times and more accurate hemostasis. 13 Major blood vessels should be ligated with the usual suture thread due to their large caliber, while those of smaller caliber can be sealed with the HS. This technique proved especially useful for removing fibrofatty tissue in the posterior triangle of the neck, as it quickly sealed smaller vessels. Manual ligation should be performed whenever there is HS failure. 5 There was a statistical difference in hemostatic capacity between both methods.

Regarding the drainage collected in the postoperative period, Walen et al., 3 Shin et al., 12 Verma et al. 5 and Fritz et al. 10 found similar results. However, Dean et al. 1 and Kos et al. 8 found a reduction in the amount drained from the first to the second postoperative day with the use of HS. Despite this, the drain permanence time (in days) was similar in all studies. Thus, although HS has superiority in terms of intraoperative coagulation efficacy, the effect measured by the postoperative drain is like the conventional technique.

The number of ligatures with sutures was higher in the group of patients undergoing ND with the conventional technique compared to HS. On the other hand, the number of dissected lymph nodes was similar in both methods, indicating that oncological radicality is not compromised by the type of hemostasis performed.

Postoperative pain measurement denotes tissue damage caused by the method used, and no statistically significant difference was found between the two groups. The HS delivers lower temperatures than the conventional technique at the time of ligation, but this did not seem to have an impact on postoperative pain. Also, postoperative hospitalization denotes morbidity due to the procedure and method used, with no significant difference.

The main advantage of HS is that the surgical field remains bloodless, facilitating surgery and reducing operative time, avoiding ligatures or clotting for hemostasis. Additionally, there is no tissue adhesion to the instrument during direct contact or transmission of electricity. As there is no stimulation to nerves or muscles, postoperative discomfort is lower. As the scalpel is used for both dissection and coagulation, there are fewer instrument changes. The HS works at lower temperatures than laser or electrocautery, resulting in less tissue damage. 1

There are limitations among these studies regarding the calculation of blood loss, surgery calculation time, nonuniformity of the ND technique (radical and selective) within the same study, and the technique being performed at the same time with primary tumor surgeries, such as mouth, thyroid, and larynx. Patients with different lengths of surgery were included. However, patients were stratified, and, within each primary study, the same methods were applied to both groups (study and control), enabling a reliable comparison, and minimizing potential bias. Differences in surgical technique between the various teams are also an inevitable limitation, which can be overcome by means of a multicenter study.

## Conclusion

This systematic review showed there is clear evidence that the use of HS for ND significantly reduces operative time, intraoperative bleeding, volume of fluid drainage, and the number of ligatures. Furthermore, there was no difference in the time of use of the drain, number of dissected lymph nodes, pain at 24 and 48 hours and length of hospital stay. Therefore, HS is a safe and effective method for ND.
